# Co-Learning: code learning for multi-agent reinforcement collaborative framework with conversational natural language interfaces

**DOI:** 10.3389/frai.2025.1431003

**Published:** 2025-05-15

**Authors:** Jiapeng Yu, Yuqian Wu, Yajing Zhan, Wenhao Guo, Zhou Xu, Raymond Lee

**Affiliations:** Guangdong Provincial Key Laboratory of Interdisciplinary Research and Application for Data Science, Faculty of Science and Technology, Beijing Normal University-Hong Kong Baptist University United International College, Zhuhai, China

**Keywords:** multi-agent, large language model, reinforcement learning, prompting, education

## Abstract

Online question-and-answer (Q&A) systems based on the Large Language Model (LLM) have progressively diverged from recreational to professional use. However, beginners in programming often struggle to correct code errors independently, limiting their learning efficiency. This paper proposed a Multi-Agent framework with environmentally reinforcement learning (E-RL) for code correction called Code Learning (Co-Learning) community, assisting beginners to correct code errors independently. It evaluates the performance of multiple LLMs from an original dataset with 702 error codes, uses it as a reward or punishment criterion for E-RL; Analyzes input error codes by the current agent; selects the appropriate LLM-based agent to achieve optimal error correction accuracy and reduce correction time. Experiment results showed that 3% improvement in Precision score and 15% improvement in time cost as compared with no E-RL method respectively. The results indicate that integrating E-RL with a multi-agent selection strategy can effectively enhance both the accuracy and efficiency of LLM-based code correction systems, making them more practical for educational and professional programming support scenarios.

## 1 Introduction

Large Language Model (LLM)-based conversational question-answering systems, such as Chat Generative Pre-trained Transformer (ChatGPT), have become prominent deep learning networks capable of addressing a wide range of tasks—everyday inquiries to professional task solutions (Nijkamp et al., 2022; Barrault et al., 2023). They can demonstrate reasoning and planning strengths to match an autonomous agent’s definition to perceive its surroundings, make decisions, operate, and even build multi-agents to solve complex problems (Xi et al., 2023; Zhou et al., 2023). The majority of multi-agent frameworks can usually complete stationary streaming tasks using fixed prompts but are unable to select the optimal agent according to specific task content (Wang et al., 2024).

Program coding involves time-consuming professional skills due to the specific task’s requirement. Beginners often strive for code understanding but may cede programming due to the lack of guidance to resolve unforeseen errors. This study proposes a *Code-Learning* community based on an LLM multi-agent framework on code correction and annotation for efficient learning in communication with users. It uses reinforcement learning to decide which agent is required for the next step based on the input problem or the output generated by the current agent, in contrast to previous multi-agent code generation, error-correction networks based on a defined single stream (Chen et al., 2023). There are five agents responsible for different tasks: (1) Main agent supervises and exchanges information with users, (2) Correction agent revises programming, (3) Interpretation agent explains the programming logic to subsequent agents to locate incorrect codes, (4) Test agent generates correct codes, and (5) Annotation agent adds comments to the revised code for user’s understanding. These five agents communicate through conversation interfaces. The multi-agent generated by the main one is a copy with E-RL to self-improve and provide feedback to both counterparts and human users. Co-Learning uses ERNIE (Sun et al., 2021), SparkDesk (iFLYTEK, 2023), and LLaMa (Touvron et al., 2023) as base models for different agents. Code error correction with E-RL performance is evaluated by passing probability tests, single loop computation time, and the number of loops required. The annotation results are evaluated by an expert reviewer through the location, accuracy, and comprehensibility of annotations.

The aims of this study are to:

1. build a multi-agent framework based on multi-LLMs for code error correction.
2. use original error code datasets to evaluate the performance of multiple LLMs.
3. explore the possibility of reinforcement learning for a large language model-based multi-agent operating environment.
4. compare benchmark frameworks to indicate significant accuracy and operating speed improvements.

## 2 Related studies

### 2.1 Prompting with feedback

Recent research on large language models has shown that effective use of prompt words can reduce adverse output (Ganguli et al., 2023) and induce LLM to generate crucial assessments (Fang et al., 2024). Prompt engineering is a specialized study with remarkable benefits for reasoning-type tasks (Madaan et al., 2024). Reflexion (Shinn et al., 2023) pointed out that using linguistic feedback can reinforce LLM instead of weights to store the feedback text in memory and induce the large language model to make better decisions, allowing the language agent to learn by mistakes efficiently. Dialog-Enabled Resolving Agents (DEAR) (Nair et al., 2023) can improve LLM judgment in clinical medicine by simulating two agents converse with each other so that the researcher agent can process information and extract key points of the problem and the decision-maker agent integrates them from the researcher agent to judge the final output accordingly. Self-debugging (Chen et al., 2023) interprets its self-generated code, assisting LLM in identifying code errors without explicitly pointing out the errors and modifications by mimicking a rubber duck test performed by human programmers without extra instructions.

Co-Learning also includes the thinking of prompting with feedback. When the generated code fails to pass the test, Co-Learning interprets and modifies its self-generated code according to the memorized linguistic feedback. At the same time, reinforcement learning will automatically select the optimal agent for the next action based on the feedback from the current agent.

### 2.2 Multi-agent framework

Multi-agent frameworks emerged at the end of the 20th century (Bradshaw, 1997) when software engineers used Java to write multi-agents for computers to perform by splitting into small, separate tasks that allow agents to focus and cooperate with each other. At the beginning of the 21st century, Java Agent Development Framework (JADE) (Bellifemine et al., 2001; Lee, 2005) standardized the multi-agent forms based on Java, which was used in finance, trading, and journalism (Lee and Liu, 2001).

Python Agent DEvelopment (PADE), a multi-agent framework based on Python (Melo et al., 2019), can be seen as a Python implementation of JADE, which re-implements JADE’s core functionalities using Python, making it more suitable for projects that rely on Python-based environments.

LLM, led by GPT (OpenAI, 2023), showed enormous potential, making it possible to employ LLM instead of programs to create agents (Li et al., 2023). LLM’s cognitive abilities in single agents have provided a multi-agent foundation (Sumers et al., 2023). Many experiments have shown that complex, dynamic tasks can be completed by multiple large language model agents equipped with strategies and communications (Zhang et al., 2023).

Hence, Co-Learning uses a PADE framework to create agents with functions, whereas multiple LLMs are the core component of a multi-agent framework and information transfer between individual agents to achieve a dynamic workflow.

### 2.3 Reinforcement learning

Reinforcement learning (RL) is a machine learning paradigm where agents interact with environments to maximize cumulative rewards (Kaelbling et al., 1996). While traditional RL approaches, such as deep deterministic policy gradients (Lillicrap et al., 2015), have shown success in domains ranging from game-playing (Silver et al., 2016) to autonomous driving (Kiran et al., 2021), their adaptation to language models introduces unique challenges. Current RL for LLMs predominantly relies on reinforcement learning from human feedback (RLHF) that requires labor-intensive preference labeling (Schick and Schütze, 2020) or memory-augmented parameter updates (Nair et al., 2023; Chen et al., 2023), incurring substantial human and computational costs. These methods still necessitate parameter fine-tuning and Graphics Processing Unit (GPU)-intensive computations, creating hardware bottlenecks for practical deployment, especially when integrating closed-source models such as Spark API, where fine-tuning is prohibitively expensive. Our framework advances this direction through three key innovations: (1) Full automation of the evaluation-reward cycle using predefined test cases and performance metrics, eliminating human-in-the-loop requirements; (2) Parameter-free adaptation through dynamic agent selection rather than weight updates; and (3) Hardware-agnostic operation via API orchestration, enabling CPU-only execution. This differs fundamentally from prompt-based RL approaches that still require human-curated reward signals and establishes a new paradigm for lightweight RL integration with LLMs.

## 3 Methodology

### 3.1 Proposed multi-agent code correction framework (Co-Learning)

**Figure 1:**
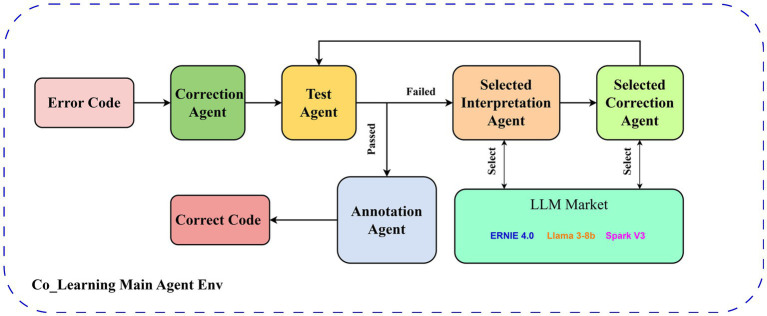
Framework of multi-agent code correction (Co-Learning).

A Co-Learning multi-agent code correction framework is illustrated in Figure 1. It has a coexistent framework that relies on PADE (Melo et al., 2019), and the entire workflow runs in the environment created by the main agent. To begin, a correction agent uses a default large language model to make an initial modification for the input error code, returns the generated sentence, and transmits it to the test agent. The test agent performs tests based on the test samples from the dataset mentioned in Section 4.1, which includes the error code and the test cases that should be passed for the code after correction. Specifically, the test agent dynamically executes the Python code generated by the correction agent and evaluates its correctness and score by running it through the basic and challenging test cases provided in the dataset. If the code passes all tests, meaning the generation is correct, the test agent will send the code to the annotation agent for annotation and output it as the correct code. If the code is unable to pass any test, the generated code will be passed to the interpretation agent, and the interpretation is stored in memory as an environmental reinforcement learning prompt. Then, an error code will transfer to the correction agent selected by reinforcement learning to re-generate a code based on the memorized code and interpretation. A loop will be formed by passing the generated result back to the test agent. Error codes entered by outsiders during actual use are not included in the test cases in the test dataset; three forms of tests used by the test agent will be provided: test samples entered by the user, test samples generated by LLM based on user-typing-requirement, and the code correctness determined directly by LLM.

For different agents, the main agent stores all hyper-parameters and historical information, using E-RL based on other agent feedback to update the state of the environment. The test agent creates namespaces to declare the generated code, uses test cases to check the generated code, and returns test results and error messages. The rest of the agents clarify their tasks according to the prompt words, combine them with historical information to generate input streams for the LLM, and return results to the main agent for storage. Co-Learning involves agents cooperating with each other, mimicking human rubber duck testing while using unit test feedback, and selecting the most appropriate large language model in a real-time manner based on E-RL to enhance the performance of code error correction.

### 3.2 Python agent development (PADE)

Python Agent DEvelopment (PADE) is a simple Python-based approach to create agents that can be accessed by different devices (Melo et al., 2019). This enables the development and creation of communication networks among different agents in accordance with the Foundation for Intelligent Physical Agents (FIPA) standards. PADE is an architecture based on Twisted to develop a multi-agent application using its library resources (Library) and perform a Running Environment of a distributed system. PADE controls the platform by creating an agent (Agent Management System) responsible for platform operations, realize for internal platform functions, and migrate agents out of the platform to other platforms.

**Figure 2:**
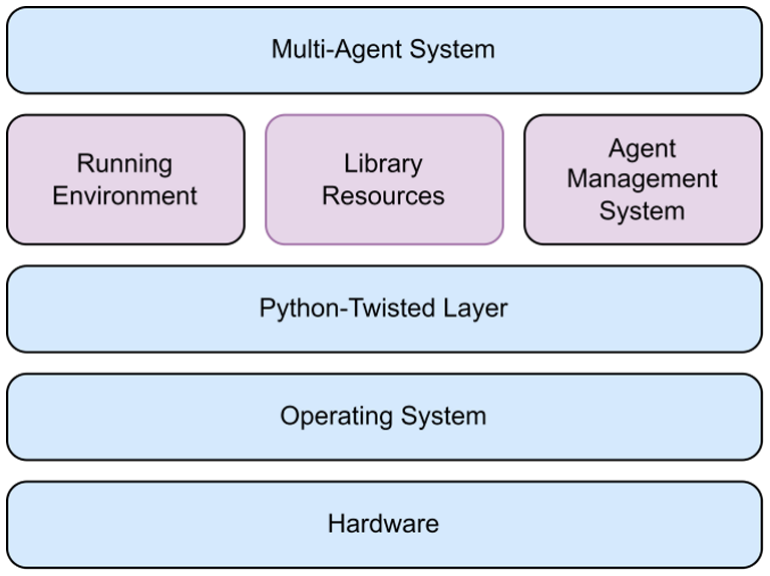
PADE architecture.

A PADE architecture is depicted in Figure 2. It consists of seven modules with the following functions:

- Core: All agents will inherit this base agent kernel framework when created;
- Behaviors: A behavioral template implemented by the agent can be inherited by the user to define a variety of personalized behaviors according to FIPA standards;
- Agent Communication Language (ACL): A language model set up (?) for information interaction between agents according to FIPA standards;
- WEB: A Web server with a graphical interface for interaction with registered sessions, agents, and message databases;
- CLI: Functions interaction with the PADE platform;
- Miscellaneous (Misc): General functions such as looping a standard form of agent initialization message on the screen;
- Tests: Module testing.

**Figure 3:**
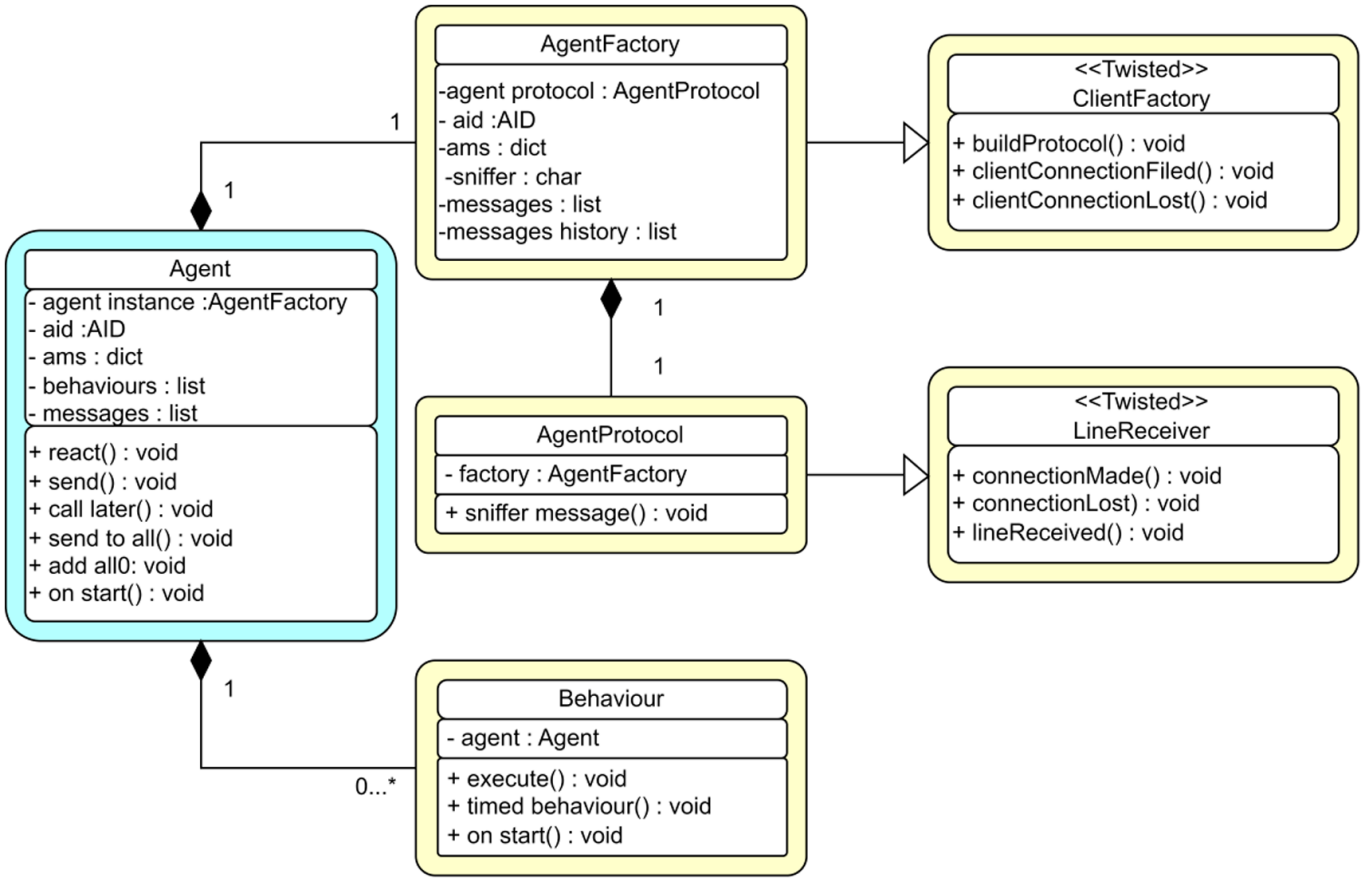
UML standard for classes in PADE framework.

The core of the PADE framework is agent execution. Figure 3 illustrates an agent execution UML structure, which indicates the class agent and its interaction with other class agents.

All agents use the PADE framework (e.g., AgentFactory, AgentProtocol in Figure 3), inherit the agent template, and conform to the Twisted protocol. They are identified by their own Agent IDentifier (AID). An agent can be seen as a connected node in the server platform network that can initiate message exchanges or respond to requests from other network nodes via their AIDs.

An Agent Management System (AMS) in PADE implements key functions such as control and supervision through a table containing AIDs of all agents according to the FIPA 00023 standards. As an agent, it is the first one to activate, and the rest of the agents are required to register and activate. Each agent in the network is required to send a message to the AMS agent so that its AID can be saved as a text string by the AMS. Then, each agent in the platform can access to a table in AMS that stores the names and addresses of all agents to identify other agents. The AMS is updated and distributed whenever an agent enters or exits the network.

**Figure 4:**
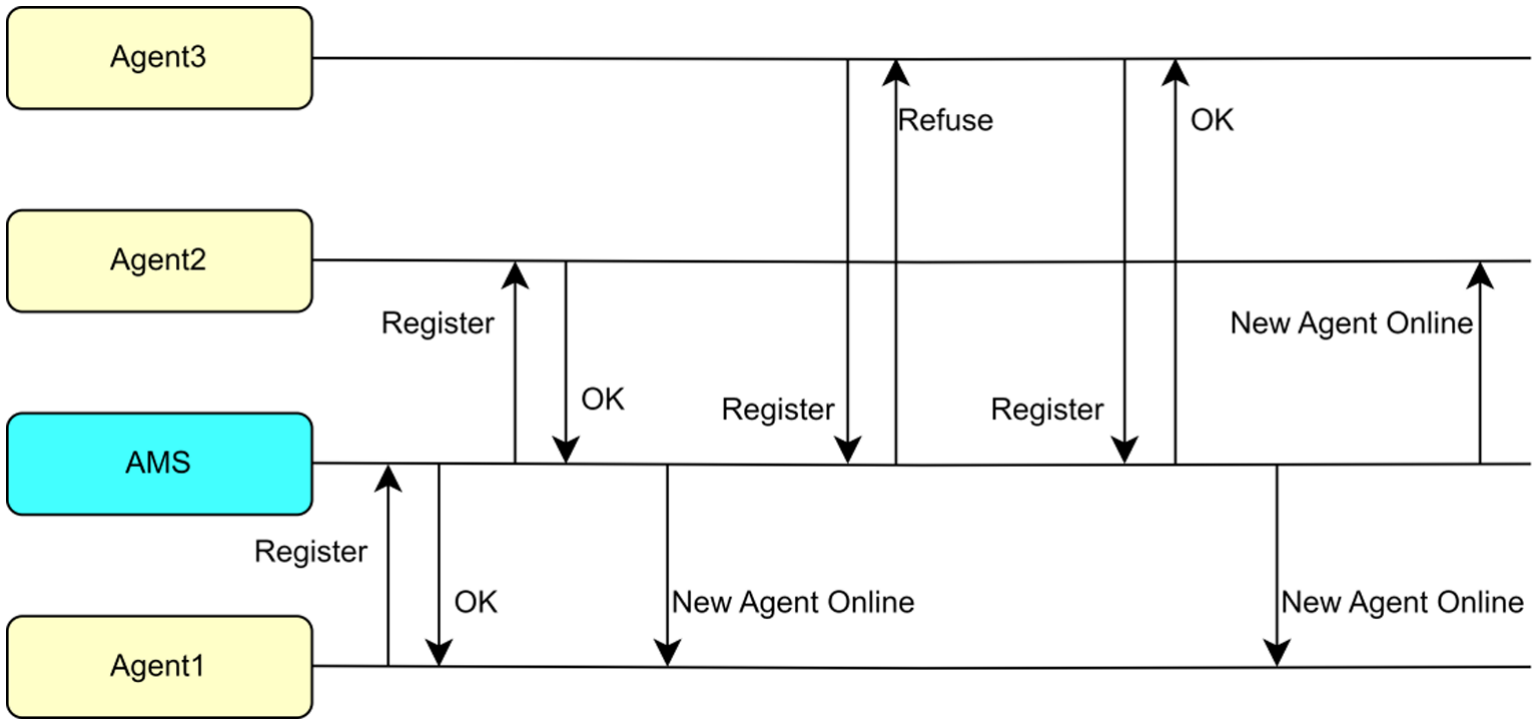
Registration and update of information in an example agent platform.

Figure 4 illustrates an example where the AMS is the first agent to be launched. It performs registration for agents 1, 2, and 3 as they enter, and informs the existing agents when a new agent joins the network. In this case, when agent 3 fails to register for the first time, its address cannot communicate with other agents. It can only send to the other agents after the second attempt is successful.

Therefore, if an agent requires communication with another agent, it will refer to the address of the target agent in its own table without asking the AMS agent. These agents can communicate even if the AMS agent is deactivated. The AMS agent can also record message interactions so that all agents in the network will send a copy of the message received to AMS each time.

AMS is the most significant network agent responsible for agent registration, monitoring, updating active agent tables, logging information exchanged among agents, sending orders to change, and deactivating agent’s behavior.

**Figure 5:**
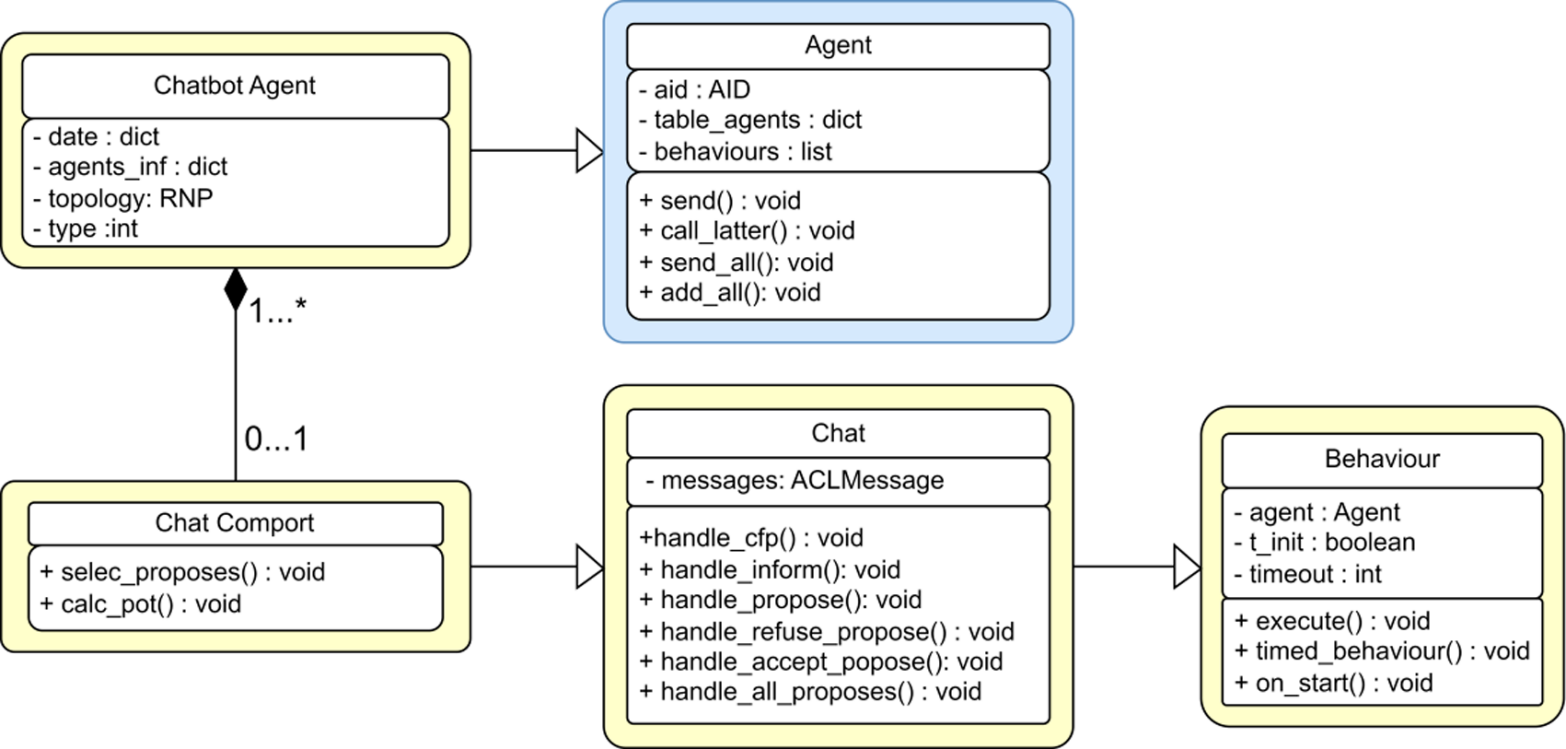
UML standard for a chat agent with FIPA chat behavior.

An agent creation has a well-defined pattern of behavioral classes defined by PADE, protocol classes, and information from the class agent provided by core. Therefore, it is common to have an example in PADE that represents a given agent, and many classes required behavior examples representation defined by each agent. Figure 5 illustrates the chat behavior in PADE. Here, the chat agent class inherits all necessary features to execute and identify the agent to communicate with other counterparts. The chat port inherits all necessary behaviors from the chat class according to FIPA standards. Moreover, the chat class has the base behavior class to implement the base startup with methods.

### 3.3 Environmentally reinforcement learning (E-RL)

The environmentally reinforcement learning (E-RL) aims to provide a structured environment for LLM-based agent, enhancing their ability to perform code error correction and testing effectively. Through E-RL, LLM-based agents select actions based on the current state $S_{\text{currect}}$ and historical context $H_{\text{history}}$ to perform tasks such as code error correction and interpretation. The interaction begins with a user-supplied code task description $T_{\text{task}}$ and error code $C_{\text{error}}$, which together form the initial state $S_{\text{initial}}$ of the system as shown in Equation 1:


(1) $$S_{\text{initial}}=(T_{\text{task}},C_{\text{error}})$$

The LLM-based agent first explains the reason for the code error, $E_{\text{error}}$, based on the current state and historical context, as follows Equation 2:


(2) $$E_{\text{error}}=f_{\text{explain}}(S_{\text{initial}},H_{\text{history}})$$

Next, the agent attempts to correct the erroneous code, $C_{\text{correct}}$, based on the generated explanation $E_{\text{error}}$ and the current state as shown in Equation 3:


(3) $$C_{\text{correct}}=f_{\text{correct}}(E_{\text{error}},S_{\text{initial}})$$

Once the corrected code is generated, it is executed and compared to two test cases with different levels of difficulty, denoted $S_{\text{test}1}$ and $S_{\text{test}2}$. The correctness of the generated code is evaluated as as shown in Equation 4:


(4) $$R_{\text{test}\;1}=f_{\text{test}}(C_{\text{correct}},S_{\text{test}\;1}),R_{\text{test}\;2}=f_{\text{test}}(C_{\text{correct}},S_{\text{test}\;2})$$

If both tests pass (i.e., $R_{\text{test}\;1}$ = 1 and $R_{\text{test}\;2}$ = 1), the interaction ends, and the final corrected code is returned as $C_{\text{final}}$ = $C_{\text{correct}}$. If either of the tests fails, the generated code has errors and will be passed to the interpretation agent for re-evaluation, and the process continues. The process forms an iterative loop, where the code correction process continues until the generated code passes all tests or the maximum number of iterations $N_{\max}$ is reached.

The discrete state space *S* in E-RL consists of historical dialogue records that encapsulate the interactions between the user and LLM-based agents, which include task descriptions, error hints, and code corrections as shown in Equation 5:


(5) $$S=\{S_{\text{initial}},S_{\text{currect}},\ldots ,S_{\text{final}}\}$$

The action space *A* consists of all possible actions that the agent can take, which are represented by natural language outputs generated by the agent based on the current state, such as code corrections $C_{\text{correct}}$, error explanations $E_{\text{error}}$, or code annotations $A_{\text{annotation}}$. We define two different reward mechanisms at the same time.

The first reward mechanism evaluates the LLM-based agent’s performance during interaction. Specifically, if the agent passes the basic test sample $S_{\text{test}\;1}$, it will be rewarded with 2 points, and if it passes the more difficult test $S_{\text{test}\;2}$, it will be rewarded with 3 points. Conversely, if the agent fails the tests, it will be penalized with 0.5 and 0.2 points, respectively. The reward function can be expressed as shown in Equation 6:


$$R_{\text{success}}=\{\begin{array}{c} \;2,\text{if fails basic test}\;(R_{\text{test}\;1}=1)\;\\ \;3,\text{if passes difficult test}\;(R_{\text{test}\;2}=1) \end{array}$$

(6) $$R_{\text{fail}}=\{\begin{array}{c} -0.5,\text{if fails basic test}\;(R_{\text{test}\;1}=0)\;\\ -0.2,\text{if fails difficult test}\;(R_{\text{test}\;2}=0) \end{array}$$

**Figure 6:**
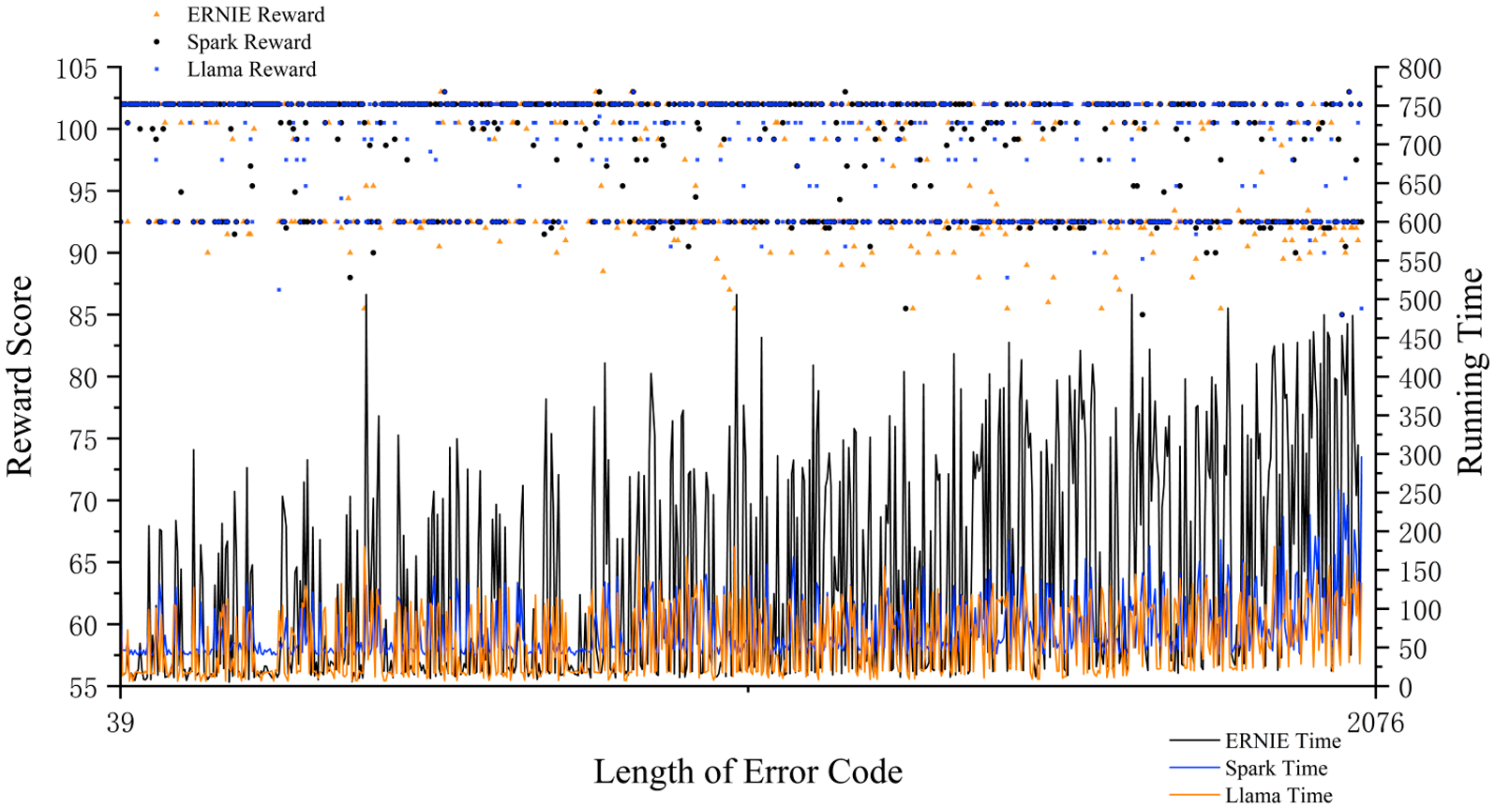
Reward scores and running time for different length of error code correction of three LLMs.

Obtaining the results in Figure 6 based on the first reward mechanism, ERNIE performs the best in terms of performance but runs the slowest, LLAMA is rated as medium in terms of performance and runs at average speed, and Spark shows the worst results although it runs the fastest. Based on these different performance characteristics, we dynamically select the initial large model based on the length of the input code. Depending on the code length, we assigned short codes to Spark, medium-length codes to LLAMA, and longer codes to ERNIE, respectively.

The second reward mechanism aims to dynamically select the applicable language model based on the performance of the LLM-based agent under the code error correction dataset. We first use *softmax* functions to obtain the weights of the three large language models on the metrics of time and reward, respectively as shown in Equation 7:


(7) $$\omega_{\text{time}}=\frac{e^{T_{i}}}{\sum_{i=1}^{k=3}e^{T_{i}}},\omega_{\text{reward}}=\frac{e^{R_{i}}}{\sum_{i=1}^{k=3}e^{R_{i}}}$$

Where $T_{i}$ is the execution time of each language model (ERNIE, LLAMA2-8B, and Spark V3) and $R_{i}$ represents the reward score for each model based on its performance in terms of code error correction tasks. The softmax function transforms the execution time and reward into normalized values, which are used as relative weights to adjust the performance of each LLM in the final decision-making process. The weights $\omega_{\text{time}}$ and $\omega_{\text{reward}}$ can be interpreted as ratios that reflect the relative importance of execution time and reward, respectively.

The reward mechanism then calculates a composite score $S_{\text{LLM}}$ for each language model based on code length $L_{\text{length}}$, run time $\omega_{\text{reward}}$, reward value R, number of loops *n* (Table 1), and stability of the language model $S_{\text{stability}}$.

**Table 1:** Number of loops required for code error correction of three LLMs.

LLM	1 loop	2 loops	3 loops	4 loops	5 loops
ERNIE 4.0	337	60	26	14	265
Llama 3-8b	317	81	32	21	251
Spark V3	319	48	14	4	317

These metrics reflect the performance and operational status of the LLM-based agent during the interaction. Then, based on the calculated composite score, the language model with the highest score is selected as the main language model in the current environment. In this way, the reward mechanism is able to automatically adjust the selected language model according to the actual performance of the LLM-based agent to improve the efficiency and accuracy of code error correction and interpretation. The algorithm used by E-RL to select LLM is showed in Algorithm 1.

**ALGORITHM 1:**
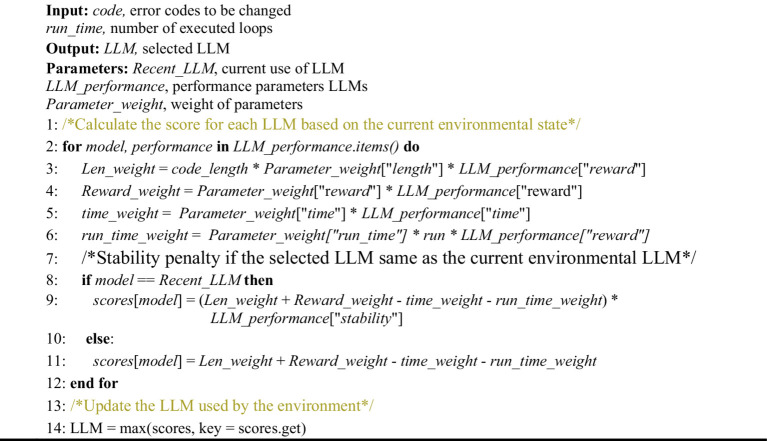
LLM options update.

## 4 Experiments

### 4.1 Data description

The dataset used in the experiments is based on the Mostly Basic Programming Problems (MBPP) test set compiled by Austin et al. (2021), which consists of Python programming problems paired with various prompting words for code generation and corresponding automated test cases. To generate an error code dataset for the Co-Learning approach, we used ERNIE-3.5-8K (Sun et al., 2021), an early model with relatively low performance compared to the models employed in Co-Learning. The ERNIE-3.5-8 K model was used to generate erroneous code based on the provided prompting words. Although the generated code is not executable, it contains correct function names and relevant comments about the code logic, making it useful for subsequent experiments. This process resulted in the creation of a new dataset consisting of 702 error codes, test samples, and challenging test cases, as detailed in Table 2.

**Table 2:** Subset of the dataset samples.

Error code	Test list	Challenge test list
def remove_Occ(string, character): … …	[’assert remove_Occ(“hello”,“l”) == “heo”’, ‘assert remove_Occ(“abcda”,“a”) == “bcd”’, ‘assert remove_Occ(“PHP”,“P”) == “H”’]	[’assert remove_Occ(“hellolloll”,“l”) == “helollol”’, ‘assert remove_Occ(“”,“l”) == “”’]
def is_woodall(number): … …	[’assert is_woodall(383) == True’, ‘assert is_woodall(254) == False’, ‘assert is_woodall(200) == False’]	[’assert is_woodall(32212254719) == True’, ‘assert is_woodall(32212254718) == False’, ‘assert is_woodall(159) == True’]

**Figure 7:**
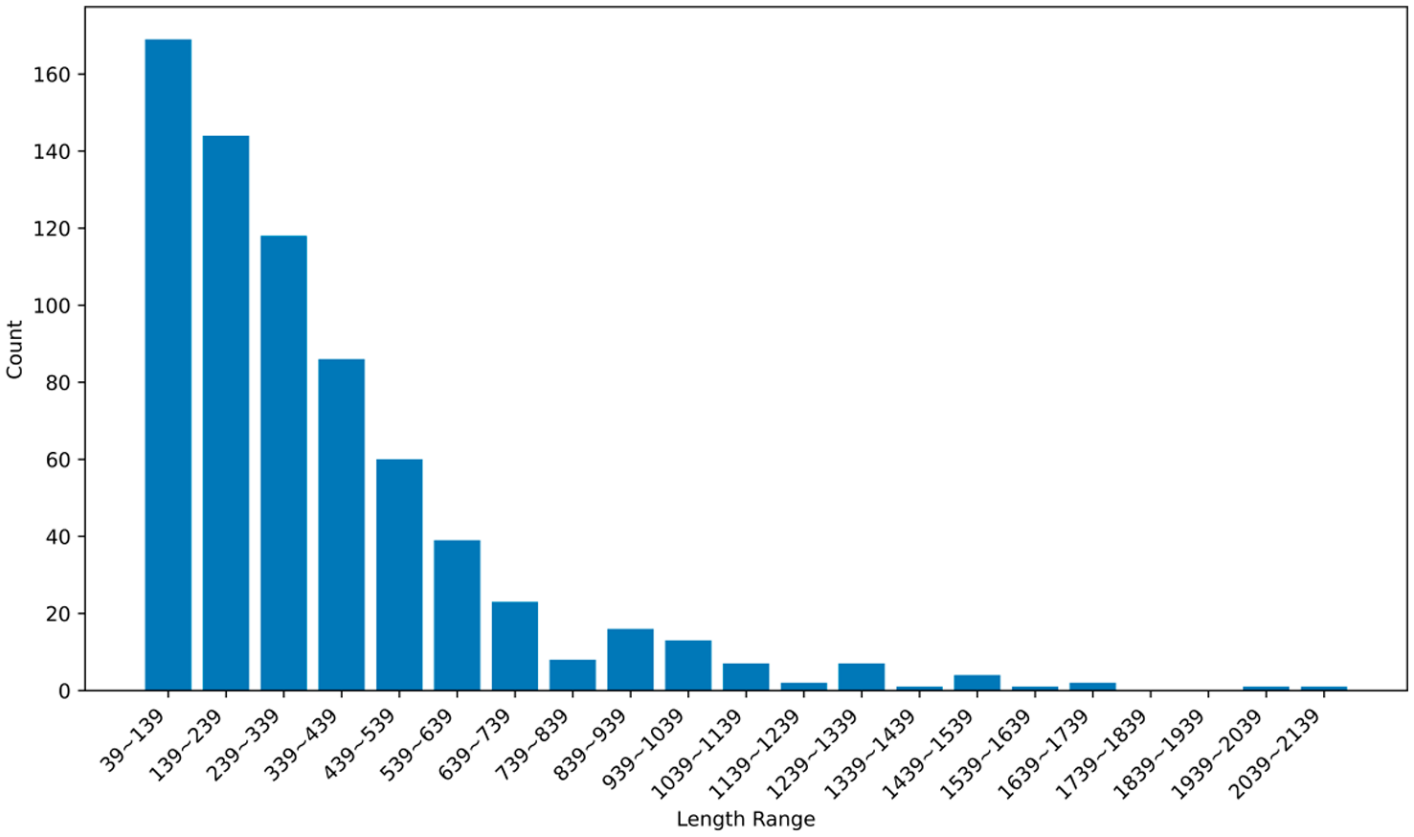
Distribution of error code length ranges.

Figure 7 presents histograms illustrating the distribution of error code lengths in the dataset. It is instrumental in selecting the best LLM for the environment at the first-time code correction. By categorizing the length of the input message, E-RL will lead Co-Learning to select more capable LLMs for the next agent when obtaining long sentences. An attempt is made to reduce the number of error correction loops while sacrificing a proportion of the generation time, thus reducing the total time consumption and avoiding unnecessary computational costs.

### 4.2 Baseline LLM

ERNIE-4.0-8 K-0329 (Sun et al., 2021), Spark Desk V3 (iFLYTEK, 2023), and Meta-Llama-3-8b (Touvron et al., 2023) are selected as the open-source LLMs. Baseline LLMs are merged into the PADE multi-agent environment. E-RL selects the optimal model for the Co-Learning framework from the three to provide high-quality responses.

### 4.3 Experimental environment

The experiments are conducted on a server with a Xeon(R) Gold 5218 CPU @ 2.30GHz (16 cores). Models are implemented in PyTorch 2.1.0 with CUDA 12.1.

### 4.4 Main results

This experiment uses the original error code dataset, sets the maximum number of cycles to 5 (exceeding the number of cycles will directly determine the operation failure), and limits the memory length to three dialogue pairs to avoid exceeding the LLM single input message length limit.

Table 3 shows the number of successfully corrected loops, average running time, and final accuracy in the code correction task using a single LLM or E-RL and a collaborative learning framework based on multiple LLMs. Co-Learning mimicked rubber-duck debugging operations can be observed to help the model retry generation when the first generation goes wrong, with Llama 3-8b being the biggest beneficiary of the single LLM model, with 134 successful re-generations of the correct code.

**Table 3:** Co-Learning with different LLM performance comparison.

Method	1 loop	2 loops	3 loops	4 loops	5 loops	Average running time (s)	Accuracy (%)
Co-Learning (ERNIE 4.0)	**337**	60	31	**29**	245	137.5	65.09
Co-Learning (Llama 3-8b)	317	81	32	21	251	112.8	64.24
Co-Learning (Spark V3)	319	48	14	4	**317**	**57.7**	54.84
Co-Learning (E-RL)	280	**104**	**65**	27	226	99.8	**67.80**

For 1-5 loops bold values represent the method that achieves the most correct results after n loops. For average running times and accuracy, bold values represent the method that achieves the best results.

Through E-RL, the probability of success of Co-Learning based on rubber duck testing is greatly increased; even if some first-time success probability is lost, a total of 196 examples are correctly modified due to E-RL. E-RL’s contribution to the runtime is also undeniable, being only slower than the high-speed, low-enabled Spark V3, with an average test time of 99.8 s. Finally, Co-Learning with E-RL has the highest review success rate, reaching 67.80%.

### 4.5 Case study

**Figure 8:**
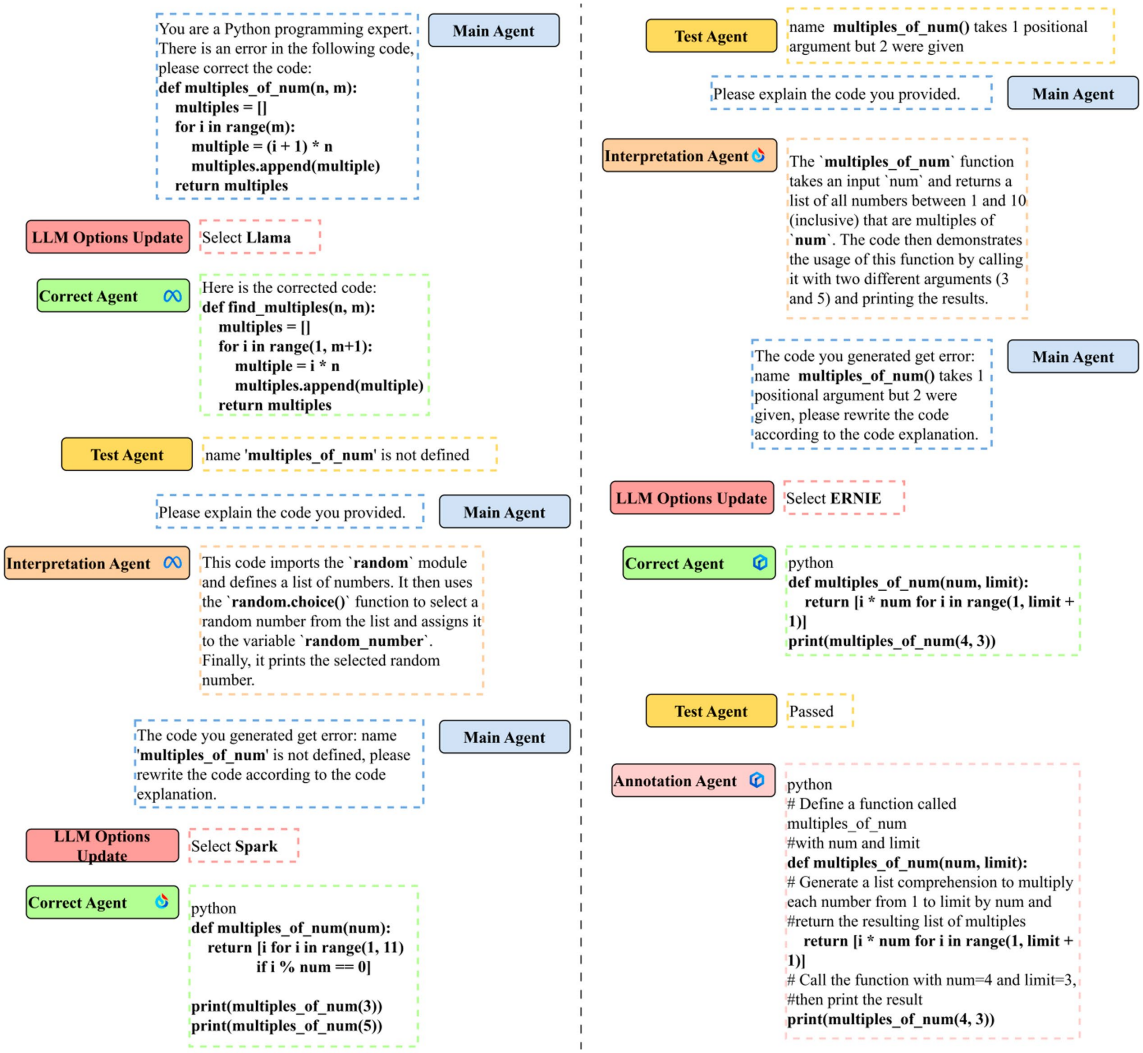
Example of Co-Learning code correction.

Figure 8 depicts the actual situation of code error correction through Co-Learning. First, the master agent schedules the error correction task, and E-RL selects Llama as the initial LLM for joint learning based on the input message. The correct agent uses Llama to make initial modifications to error codes. Based on the results, it can be concluded that Llama generated the correct answer but secretly changed the function name. This phenomenon is obvious in the three LLMs. The LLM may choose more appropriate function names for the code based on the code content, while ignoring the user’s needs.

The test agent detects that the generated code fails the test and returns an error message to the main agent. The main agent assigns code interpretation tasks in the hope of simulating rubber duck testing. The interpretation agent interprets the generated code and stores the contents in the main agent memory. E-RL re-selects Spark as the next agent’s LLM, and the correct agent then re-corrects the code. Spark misinterprets the code as outputting multiples of the current number between 1 and 10, which may be related to the fact that the error returned by the test agent contains information about the first test sample, where the maximum number to be generated is 10.

The test agent detected the error, and the leading main agent decide to make another rubber duck debugging. Then, LLM was changed to the most powerful ERNIE, which eventually generated the correct code, and the annotation agent added comments to the output.

This case shows how Co-Learning can continuously correct, understand, and then correct erroneous code by imitating the error correction process of human programmers, and generate information-intensive integrated code that only senior programmers can generate. With E-RL, Co-Learning attempts to balance model power and speed in the hope of generating the best response in the shortest possible time, creating code that is shorter and more refined than expected correct code, while using less time than a single model.

## 5 Conclusion and future works

This study focuses on developing a code learning community (aka Co-Learning) framework based on an LLM-based multi-agent framework that leverages ambient reinforcement learning (E-RL) for agent self-improvement. The community aims to interpret error codes and perform code correction tasks to provide users with a more intelligent and personalized programming learning experience. Experiments show that the Co-Learning framework can effectively improve the code error correction capabilities of current LLM. E-RL dynamically determines the state of the environment and changes the selection of the LLM, which can speed up the code correction process and achieve significant improvements in the quality of the generated output.

In the future, Co-Learning will focus on further optimizing the E-RL algorithm to improve the agent’s learning efficiency and performance. At present, it seems too simplistic to select E-RL parameters based only on model capabilities. Making Co-Learning’s environmental reinforcement learning have dynamic self-updating weights by combining machine learning will be one of the main goals in the future; Expect to explore more complex tasks and scenarios, including error correction and code understanding in larger code bases, as well as code learning in different programming languages and domains.

## Data Availability

The raw data supporting the conclusions of this article will be made available by the authors, without undue reservation.
